# A nonparametric approach for determining significance of county cancer rates compared to the overall state rate: illustrated with Minnesota data

**DOI:** 10.1007/s10552-012-9920-2

**Published:** 2012-04-11

**Authors:** Alan P. Bender, Allan N. Williams, John Soler, Margee Brown

**Affiliations:** Minnesota Department of Health, 85 East 7th Place, PO Box 64882, St. Paul, MN 55164-0882 USA

**Keywords:** Cancer, Counties, Significance, Surveillance

## Abstract

**Background:**

The study of the geographical distribution of disease has expanded greatly with GIS technology and its application to increasingly available public health data. The emergence of this technology has increased the challenges for public health practitioners to provide meaningful interpretations for county-based state cancer maps.

**Methods:**

One of these challenges—spurious inferences about the significance of differences between county and overall state cancer rates—can be addressed through a nonparametric statistical method. The Wilcoxon’s signed rank test (WSRT) has a practical application for determining the significance of county cancer rates compared to the statewide rate. This extension of the WSRT, developed by John Tukey, forms the basis for constructing a single confidence interval for all differences in county and state directly age-adjusted cancer rates. Empirical evaluation of this WSRT application was conducted using Minnesota cancer incidence data.

**Results:**

The WSRT procedure reduced the impact of statistical artifacts that are frequently encountered with standard normal significance testing of the difference between directly age-adjusted county and the overall state cancer rates.

**Conclusion:**

Although further assessment of its performance is required, the WSRT procedure appears to be a useful complement for mapping directly age-adjusted state cancer rates by county.

## Introduction

### The county as a geographic basis for describing state cancer incidence

For many years, investigators have evaluated geographic patterns of cancer occurrence by county to generate etiologic hypotheses [1–3]. For example, observations of higher lung cancer and mesothelioma rates in the shipbuilding areas on the eastern seaboard of the United States contributed to an understanding of the carcinogenic potential of asbestos [4]. Cancer rates in counties with nuclear power plants have been compared to similar counties without plants to address public concerns [5]. In Minnesota, the observation of a large and persistent increase in mesothelioma incidence in the northeastern region of the state led to the discovery that taconite miners were at high risk of the disease [6].

Evaluation of cancer rates by county for etiologic hypothesis development and testing has limitations [7, 8]. These evaluations are ecologic analyses [9] that are based on aggregated exposure and demographic data for larger areas and frequently diverse populations. The emergence of the analytic capabilities of geographic information systems (GIS) has led many investigators to study geographical variations of cancer occurrence for geographic units smaller than the county [10].

Displaying cancer incidence by county remains a valuable tool for describing the occurrence of cancer to the general public. County cancer rates are also important to community health assessments and allocation of public health resources [11]. County administrators and local public health officials are keenly interested in knowing how their county’s cancer rates compare to the state and whether any differences are significant. A wide range of audiences identify with county-based cancer rates, and for the purpose of communicating with these audiences, the county is often the geographic unit of choice [12, 13].

### Challenges of disease mapping

In 1976, the eminent statistician Tukey [14] described the future challenge for disease mapping as the requirement to inform and not mislead through the creation of what he called a “propaganda device.” Today, the development of GIS has expanded the capacity to map large databases of health outcomes at a high level of spatial specificity. These data can now be linked to other environmental information and population risk factors [15]. The challenges articulated by Tukey 35 years ago continue to apply to the spatial measurements and statistics of GIS analysis today [16].

The appropriate methodology to ensure that maps of health statistics inform and not mislead is the subject of much discussion and research [17, 18]. Statistical inaccuracies often arise from mapping differences between county and the overall state directly age-adjusted cancer rates. Inaccurate specification of significance when multiple counties and the state are compared, and inaccuracy of estimates used in calculating standard normal tests of significance can lead to spurious inferences about the number of statistically significant differences between county and overall state cancer rates.

### Multiple comparisons

Developing a map representing differences between county rates and the overall state rate involves many different geographic comparisons (eighty-seven in Minnesota). A table of eighty-seven differences provides the opportunity for simultaneously evaluating eighty-seven separate hypotheses. If there is a 5% chance that each of the individual confidence intervals does not contain the true difference in rates, the probability that all eighty-seven confidence intervals contain the true difference is considerably less than 95%. The distinction between a single statistical test of hypothesis that the county rate and state rate are not different and a test of all differences is referred to as the multiple inference or multiple comparison problem [19].

When the number of comparisons is large, the potential for error in the inferences increases. In Minnesota, the probability that at least one of the differences will be found to be statistically significant (at the 0.05 level) is 1 − (0.95)^87^ = 0.99 [20]. Comparing many county rates creates a markedly greater probability of generating positive findings due to chance than does the stated 0.05 level.

The problem of multiple comparisons applies to cancer mapping as well. The graphical representation is based on the tabular values of differences between county and overall state rates. If the comparisons are mapped based on a single comparison test of statistical significance, then eighty-seven different comparisons are implied but not distinctly stated by the map itself. The high likelihood that one or more of the “statistically” different county rates is due to chance is integrated into the global representation of the map.

### Errors in estimation

The standard normal statistical test for the difference between two directly age-standardized cancer rates can be approximated by a formula that assumes that the covariance of the two rates is zero, that is, the two rates are independent. The estimate for the variance of the difference between two directly age-adjusted cancer rates is the sum of each cancer rate’s variance estimate [21]. If the independence assumption is violated, a more accurate representation for the variance of the difference in rates is the sum of the two variances minus twice their covariance [22]. As the number of tests of significance increases, the possibility of major violations of the underlying assumption of independence also increases.

The assumption of independence is frequently violated when one of the rates is for a populous county. Cancer rates for populous counties are often positively correlated with the overall state rate since the county has a relatively large impact on the state rate. Assuming that the covariance is zero, when it is numerically positive, creates a loss of statistical power (the confidence intervals are spuriously large). Removing the county before calculating the overall state rate would mitigate this dependence to some degree but would result in a different overall state rate for each county—an undesirable outcome.

A negative covariance can occur for sparsely populated rural counties. For example, if populous urban counties had a higher smoking prevalence (compared to some non-urban counties) as in Minnesota, then the overall state rate for smoking-related cancers would tend to be higher and the cancer rates for counties with lower smoking prevalence would be relatively lower for these cancers. The result of assuming that the numerically negative covariance is zero would be an over specification of significance (spuriously narrow confidence intervals).

This paper describes a method to evaluate the significance of county cancer incidence rates compared to the overall state rate that is more resistant to the artifacts created by these statistical inaccuracies than are the frequently used standard normal tests of significance. This method is complementary to the graphical presentation of county cancer rates employed by states and larger governmental units when specification of significance is also desired.

## Methods

Tukey’s modification of the Wilcoxon’s signed rank test (WSRT) is a nonparametric procedure. A detailed description of the theoretical basis of the large sample approximation for the distribution-free confidence interval employed here is given in the text [23]. This text is still considered by many statisticians as a standard for applied nonparametric statistics [24].

The WSRT procedure as applied here uses a measure of the average of all possible boundaries of the *m* (number of counties) 95% standard normal confidence intervals. Six steps based on Tukey’s extension of the WSRT procedure are proposed to calculate a joint confidence interval for all differences between directly age-adjusted county cancer rates and the overall state rate.

- Form *m* 95% standard normal confidence intervals using the Keyfitz formula [25] for the standard errors of the directly age–adjusted rates creating the *m* lower and upper confidence limits: (CIL_*i*_, CIU_*i*_); *i* = 1, *m*.
  Let diff_*i*_ = *c* rate_*i*_ − *s* rate; *i* = 1, *m* (*c* is the county, *s* is the state), and se_*i*_ = standard error of *c* rate_*i*_, is calculated as follows, for a given sex and 18 age groups, ${\text{se}}_{i}^{2} = \sum\nolimits_{j = 1}^{18} {\left( {N_{j} /P_{j}^{2} } \right)(W_{j} )^{2} ;}$ where *N*_*j*_ is the number of specific cancers (e.g., lung) in the *i*th county, *P*_*j*_ is the population of the *j*th age group for the given sex of the *i*th county, *W*_*j*_ is the US 2000 standard population weight for the *j*th age group, se = standard error of s rate, (calculated with total state data for *N*_*j*_ and *P*_*j*_) and, the standard error of the difference between the county and state rate, ${\text{se}}\,({\text{diff}}_{i} ) = \left( {{\text{se}}_{j}^{2} + {\text{se}}^{2} } \right)^{1/2}$, with the 95% standard normal confidence interval = diff_*i*_ ± 1.96 se(diff_*i*_) = (CIL_*i*_, CIU_*i*_) [21].
- Form the $M = \frac{m(m + 1)}{2}$ averages of the lower and upper bounds of the *m* 95% standard normal confidence intervals [23]. These are called Walsh (*W*) averages.
  For *m* = 87 (Minnesota), $$ M = \frac{m(m + 1)}{2} = 3, 828, $$
  $$ W_{L}^{k} = ({\text{CIL}}_{i} + {\text{CIL}}_{j} )/2, $$
  $$ W_{U}^{k} = ({\text{CIU}}_{i} + {\text{CIU}}_{j} )/2;\quad i \le j,\;k = 1,2, \ldots ,3,828. $$
- Rank (sort) both lower and upper sets of Walsh averages, so that:
  $$ W_{L}^{1} \le W_{L}^{2} \le \cdots \le W_{L}^{M} , $$
  $$ W_{U}^{1} \le W_{U}^{2} \le \cdots \le W_{U}^{M} . $$
- The 1 − α confidence interval for the lower limit is $\left( {W_{L}^{C\alpha } ,W_{L}^{(M + 1 - C\alpha )} } \right)$, and the 1 − α interval for the upper limit is $\left( {W_{U}^{C\alpha } ,W_{U}^{(M + 1 - C\alpha )} } \right),$ where $C\alpha = \frac{m(m + 1)}{4} - Z_{(\alpha /2)} \left[{\frac{m(m+ 1)(2m + 1)}{ {24}}}\right]^{1/2}.$
  (*Cα* rounded to the nearest integer determines the two elements of the ordered arrays of Walsh averages that serve as end points of the joint confidence interval, α is usually 0.05).
- For *i* = 1, *m* use $\left( {W_{L}^{C\alpha},  \quad W_{U}^{(M + 1 - C\alpha )} } \right)$ as the joint confidence interval for all the diff_*i*_.
- If the (CIL_*i*_, CIU_*i*_) does not contain zero, the significance of the diff_*i*_ is then determined by the joint confidence interval derived in 5).
- If the diff_*i*_ lies outside the joint confidence interval, it is concluded that the county rate is different than the state rate. For *m* = 87 counties and *Z*_(α/2)_ = 1.96, *C*_*α*_ = 1,451, *M* + 1 − *C*_*α*_ = 2,378; and 5) becomes $\left( {W_{L}^{1451}, \quad W_{U}^{2378} } \right)$.

A detailed example of the calculations required for the application of this nonparametric procedure described by steps (1)–(6b) is given in “Appendix 1.” This algorithm produces a joint (single) confidence interval for all differences between the county and state rates, for a given sex and cancer. The 95% standard normal confidence intervals, (CIL_*i*_, CIU_*i*_), are specific to each county for the null hypothesis H_0_: diff_*i*_ (county rate_*i*_—state rate; for a given sex and cancer) = 0.

The purpose of the analysis is to identify differences in cancer rates that lie outside (CIL_*i*_, CIU_*i*_) whose statistical significance is less likely due to multiple comparisons and possible lack of the independence of the two rates. Therefore, the application of step 6b) requires that step 6a) first be satisfied. Any difference where zero lies inside the standard normal 95% confidence interval is judged as nonsignificant and is not compared to the joint interval $\left( {W_{L}^{C\alpha} ,\quad W_{U}^{(M + 1 - C\alpha )} } \right)$.

The number of years, age groups, number, and types of cancer were varied, and the performance of the WSRT procedure was evaluated for 300 separate analyses of different combinations of these variables for Minnesota and its eighty-seven counties. The performance was evaluated by the empirical method of comparing inferences derived from the WSRT procedure to those derived from the 95 and 99% standard normal tests and the 90% Bonferroni test of significance (the Bonferroni test is based on the standard normal test of significance with the significance level adjusted to reduce the number of false positives) [26]. Specificity of the WSRT procedure was assessed by determining if an elevated number of significant differences were reduced. Sensitivity was evaluated by determining which of the discrepant inferences derived from the three tests and the WSRT procedure were valid, based on a detailed examination of additional data.

The data used for these comparisons were from the Minnesota Cancer Surveillance System [27]. The MCSS is a population-based statewide cancer registry that has been in operation since 1988. Several examples of these analyses are provided to illustrate the performance of the WSRT procedure to this application. Results of the WSRT procedure are labeled in the parlance suggested by Tukey as, “unusually low,” “not unusual,” and “unusually high” [14].

## Results

**Fig. 1:**
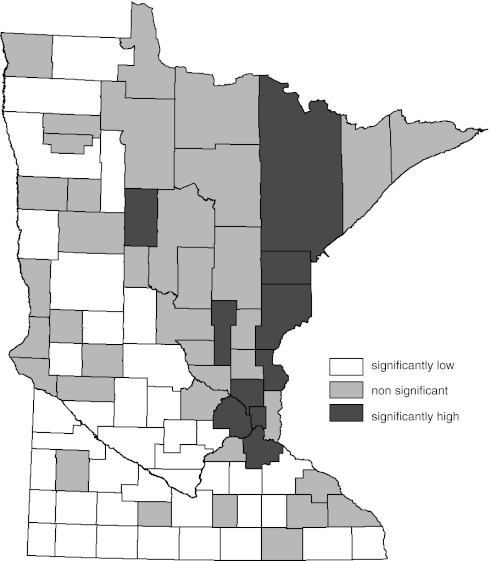
Age-adjusted female lung cancer incidence compared to overall state female age-adjusted rate by county (1988–2007). Significance determined by 95% standard normal confidence interval for difference in the two rates

Figure 1 is a map of the 95% standard normal significance tests of the difference between the directly age-standardized, county-specific female lung cancer incidence rates for 1988–2007 and the corresponding overall Minnesota rate [21, 25]. The number of counties with significantly low lung cancer rates is the dominant feature of this map.

**Fig. 2:**
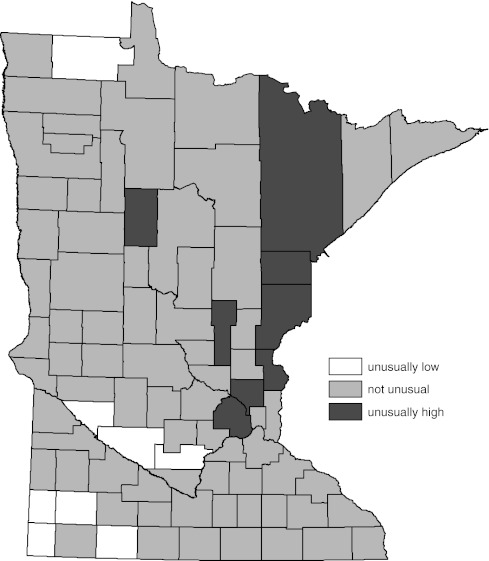
Age-adjusted female lung cancer incidence compared to overall state female age-adjusted rate by county (1988–2007). Significance determined by Wilcoxon’s signed rank test (WSRT) procedure described in text

Figure 2 contains the WSRT procedure alternative to the map provided by Fig. 1. The difference between Figs. 1 and 2 is the method used to determine which of the county rates were different than the overall state rate. The WSRT procedure determination of significance generated a map (Fig. 2) in which the number of counties with significantly different rates was substantially reduced.

The National Cancer Institute’s SEER*Stat software provides additional parametric methods, based on rate ratios, for determining statistical significance of county age-adjusted cancer rates compared to the overall state rate [28]. The results of the SEER*Stat determinations of significance, and the standard normal methods evaluated in this report, for the data depicted in Figs. 1 and 2 are given in Table 1. Results of the standard normal tests and the SEER*Stat rate ratio tests of significance produced similar results. For relatively large counts, this similarity is expected [29, 30] and these methods would result in a map similar to Fig. 1.

**Table 1:** Comparison of significance assessments of age-adjusted female lung cancer incidence compared to overall state female age-adjusted rate by county (Minnesota, 1988–2007)

Significantly low						Significantly high						Nonsignificant					
SN95	SN99	B90	T95	F95	WSRT	SN95	SN99	B90	T95	F95	WSRT	SN95	SN99	B90	T95	F95	WSRT
*Summary counts for the 87 counties*																	
40	25	18	35	34	8	10	7	4	10	10	8	37	55	65	42	43	71

Standard Normal 95% CI (SN95), Standard Normal 99% CI (SN99), Bonferroni 90% CI (B90), Tiwari 95% CI (T95) [30], Fay 95% CI (F95) [29], and WSRT 95% CI (WSRT) [Wilcoxon’s signed rank test—see text]

Table 2 contains a summary for all MCSS data, 32 cancer types (“Appendix 2”), 20 years (1988–2007), and all age groups that represented 429,794 cancers. Biologically, inconsistent cancer and gender combinations were excluded. There were five of these. The number of total comparisons for the 1988–2007 aggregated data was 5,133 (27 × 87 × 2 + 5 × 87).

**Table 2:** Number (%) of statistically significant county rates for four tests of significance—32 cancers, 87 counties, and 2 sexes

Standard normal (95%)		Standard normal (99%)		Bonferroni (90%)		WSRT^a^ (95%)	
Low	High	Low	High	Low	High	Low	High
882 (17.2)	170 (3.3)	616 (12.0)	69 (1.3)	488 (9.5)	34 (0.7)	130 (2.5)	76 (1.5)

Total comparisons = 5,133. Biologically inconsistent cancer and gender combinations excluded

MCSS 1988–2007 [All ages (*n* = 429,794 total cancers)]

^a^Wilcoxon’s signed rank test—see text

The 95% standard normal test resulted in 882 (17.2% of 5,133) differences between county and overall state rates being classified as significantly lower than zero and 170 (3.3%) as significantly larger than zero. In other words, there were 882 county rates significantly lower than the overall state rate and 170 significantly higher. This large proportion of significantly low rates is a measure of the spuriously significant differences that can result when comparing many county rates to the overall state rate and when the independence of the county rates and the overall state rate are an issue. Another indication of this artifact is provided by the 170 comparisons that were significantly high. Only 128 (2.5%) of the rates were expected to be significantly high.

Results of the 99% standard normal test demonstrated the expected overall reduced numbers of statistically significant results. However, there was still evidence of spuriously significant differences, most notably for low rates (12% significant). The 90% Bonferroni test, designed to reduce the number of false positives, was more conservative, in that, it severely reduced the number of significantly elevated rates to 0.7%. The Bonferroni test was not as effective in mitigating the impact of low rates; 488 (9.5%) remained significant.

Results from the WSRT procedure provided a different perspective. Only 2.5% of the county rates were significantly low and 1.5% were high. The number of significantly low rates was reduced, and the sensitivity for detecting elevated county rates did not appear to be greatly impaired. The 1.5% of the rates considered significantly high exceeded that estimate for both the 99% standard normal and the 90% Bonferroni tests. The only result that was larger (3.3%) was for the 95% standard normal test and that number was likely skewed too large by the multiple comparisons (5,133) that were made. For these data, the WSRT procedure greatly reduced the number of apparently spurious results generated by the three standard normal tests of significance.

**Fig. 3:**
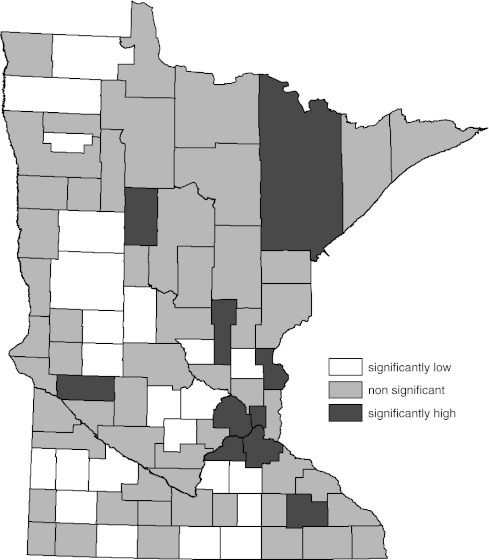
Age-adjusted female all-cancer incidence compared to overall state female age-adjusted rate by county (1988–2007). Significance determined by 95% standard normal confidence interval for difference in the two rates

**Fig. 4:**
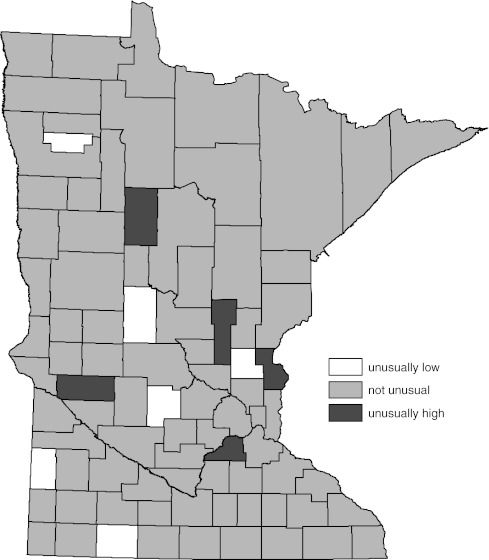
Age-adjusted female all-cancer incidence compared to overall state female age-adjusted rate by county (1988–2007). Significance determined by Wilcoxon’s signed rank test (WSRT) procedure described in text

Figures 3 and 4 represent the comparison of the female age-adjusted all-cancer incidence rate to the state rate for the years 1988–2007 by county. Determination of significance in Fig. 3 was by the 95% standard normal test and in Fig. 4 by the WSRT procedure. The difference in impressions of significance created by eliminating statistical artifact afforded by the WSRT procedure compared to the standard normal tests is seen again in these two figures. Throughout this evaluation, reduction in the number of counties with cancer rates statistically different than the overall state rate was a prominent feature of the WSRT procedure.

### Large numbers

Precision of the estimates for age-adjusted rates mapped in Figs. 3 and 4 was relatively high. These rates included all female cancers, for all years, which incorporated the largest numbers available. The problem of estimating rates from a small number of occurrences, or small populations, is well documented [31]. Large numbers represent another problem [32] that frequently results in specification of statistical significance for very small differences in rates.

**Fig. 5:**
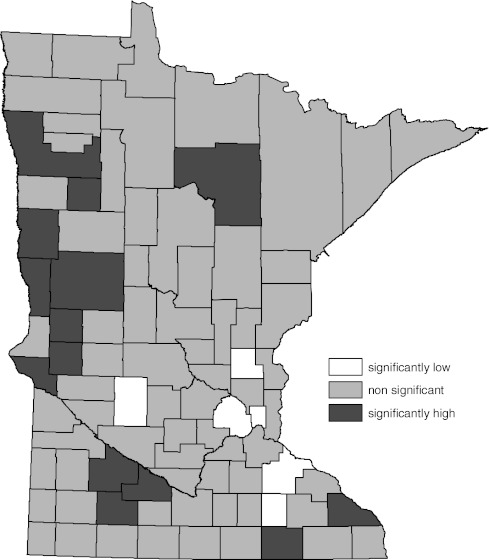
Age-adjusted male colon/rectum cancer incidence compared to overall state male age-adjusted rate by county (1988–2007). Significance determined by 95% standard normal confidence interval for difference in the two rates

**Fig. 6:**
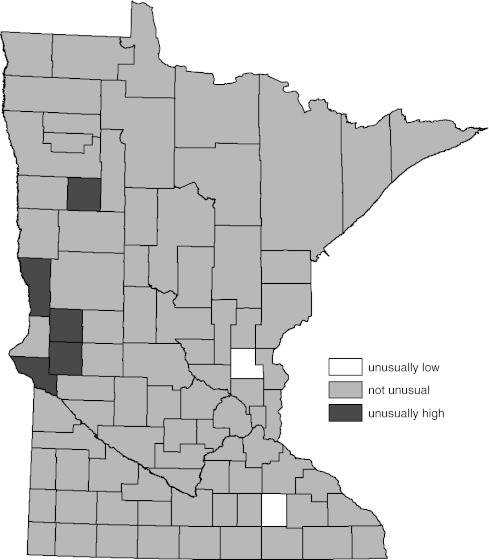
Age-adjusted male colon/rectum cancer incidence compared to overall state male age-adjusted rate by county (1988–2007). Significance determined by Wilcoxon’s signed rank test (WSRT) procedure described in text

Figures 5 and 6 represent comparisons of the incidence of a common male cancer (colon/rectum) to the overall state rate for the years 1988–2007 by county. Results of the 95% standard normal significance determinations yielded 6 (6.9%) counties with low rates and 14 (16.1%) with significantly higher age-adjusted rates than the overall Minnesota rate. The WSRT analysis yielded 2 (2.3%) and 5 (5.7%) of the counties with unusually lower and higher rates, respectively, than the cancer rate for the state as a whole. For this example, the WSRT procedure reduced the number of significant outcomes by nearly two-thirds. The WSRT procedure consistently reduced the number of significant inferences for differences between county and overall state rates when the comparisons involved relatively common cancers (large numbers).

### Small numbers

For less common cancers, there were many zero occurrences in a county during the time period (1988–2007). Of the 882 (Table 2) county rates significantly lower than the corresponding state rate, 358 of these were for zero rates. Of these 358, 339 were considered nonsignificant deviations from the overall state rate by the WSRT procedure; only 19 of 130 low rates were derived from zero county rates. The relative insensitivity to the impact of zero occurrences (and very small cell size in general) is a known behavior of the WSRT procedure [23] that is important to reducing small number artifact that complicates comparison of individual county cancer rates to the overall state rate.

Table 3 contains similar data as Table 2 except the years included were 2003–2007. The total number of cancers was reduced from 429,794 to 121,908 and the number of zero occurrences in the counties increased. The effect of this increase was to sharply elevate the number of significantly low rates derived from the standard normal tests. The number of significantly low rates identified by the WSRT procedure decreased from 130 (2.5%) to 82 (1.6%). This decrease was a desirable outcome. As the precision of the estimates decreased (with less nonzero data), the number of occurrences considered significantly low should also decrease. As seen with the more numerous data from Table 2, the sensitivity of the WSRT procedure for elevated rates, determined by the percentage of comparisons that were significantly high, was between that of the 95% standard normal test and the other two methods.

**Table 3:** Number (%) of statistically significant county rates for four tests of significance—32 cancers, 87 counties, and 2 sexes

Standard normal (95%)		Standard normal (99%)		Bonferroni (90%)		WSRT^a^ (95%)	
Low	High	Low	High	Low	High	Low	High
1,256 (24.5)	85 (1.7)	1,094 (21.3)	27 (0.5)	943 (18.4)	7 (0.1)	82 (1.6)	38 (0.7)

Total comparisons = 5,133. Biologically inconsistent cancer and gender combinations excluded

MCSS 2003–2007 [All ages (*n* = 121,908 total cancers)]

^a^Wilcoxon’s signed rank test—see text

Table 4 contains data for childhood (ages 0–14 years) cancers for all years combined (1988–2007). This analysis was based on a substantially smaller number of cancers (3,188) than Table 3. Since all 32 cancer types were analyzed separately, there were a very large number of zero occurrences even for larger counties. This created an extreme situation for data populated with zero or a very small number of occurrences. The performance of the WSRT procedure in reducing the number of spuriously low rates was even more evident in this extreme situation.

**Table 4:** Number (%) of statistically significant county rates for four tests of significance—32 cancers, 87 counties, and 2 sexes

Standard normal (95%)		Standard normal (99%)		Bonferroni (90%)		WSRT^a^ (95%)	
Low	High	Low	High	Low	High	Low	High
1,990 (38.8)	3 (0.01)	1,813 (35.3)	0 (0.0)	1,728 (33.7)	0 (0.0)	9 (0.2)	0 (0.0)

Total comparisons = 5,133. Biologically inconsistent cancer and gender combinations excluded

MCSS 1988–2007 [Ages 0–14 (*n* = 3,188 total cancers)]

^a^Wilcoxon’s signed rank test—see text

### Example: bone cancer

For the period 2003–2007, there were 168 bone cancers diagnosed in males and 113 in females of all ages in Minnesota. The overall state male standard normal 95% confidence interval (CI) for the incidence rate per 100,000 per year was (1.1, 1.6) and the female 95% CI was (0.7, 1.0). By this measure, any county with a zero occurrence had a statistically significant low bone cancer incidence. A zero occurrence resulted in a zero estimate for the county standard error that reduced the 95% CI for the difference between the county and the overall state rate to that of the 95% CI for just the state rate.

There were 31 counties with zero occurrences for males and 50 counties with zero occurrences for females. Thus, 81 (46.6%) of the 174 comparisons were considered as significantly low by the 95% standard normal test of significance. None of the counties had significantly elevated rates. (Another 59 counties had one or two occurrences, all of them not statistically different than the overall state rate). The results for the 99% standard normal and the 90% Bonferroni significance tests were identical to the 95% standard normal test; 81 (46.6%) were significantly low, and none were significantly elevated.

These evaluations could be used in two ways to create a cancer map. Maps (for males and females separately) portraying almost half of the state’s counties as having significantly low rates of bone cancer could be created. Alternatively, a decision could be made that, except for a small number of larger counties, the rates were too unstable to warrant mapping cancer rates by county.

Both of these approaches would be problematic. The first set of maps would create a misrepresentation of significance. The second approach would not take advantage of the county-specific data on cancer incidence and the composite information available from these observations.

The WSRT procedure identified none of the county bone cancer incidence rates as unusually low or unusually high. Utilizing all the data on bone cancer incidence, there was no evidence of an unusual rate of this cancer for the period 2003–2007 in any of the counties. Two maps could be created that would provide the impression that there were no unusual occurrences of male or female bone cancer incidence for the period 2003–2007 in any of the counties. This would be the most appropriate representation of the MCSS data and the one supported by the WSRT procedure.

### Sensitivity

Sensitivity for detecting unusual occurrences of cancer, especially for childhood cancers, is important. In Table 4, there are three occurrences that were statistically elevated based on the 95% standard normal significance test, but were nonsignificant based on the WSRT procedure. These occurred in Stearns County (a large county located just northwest of the Minneapolis—St. Paul area). The three occurrences were due to the high childhood female acute lymphoblastic leukemia (ALL) rates. The high rate of childhood female ALL increased both the categories of “all leukemia” and “all cancer” rates for females to bring the total elevated to 3.

The ALL data for Stearns County were examined by age groups (0–4, 5–9, and 10–14) to evaluate the consistency of this elevated rate. None of the rates for males were statistically significant based on the 95% standard normal test. ALL rates for females were consistently higher than the state rate. For the period 1998–2002, the difference reached statistical significance: 12.2 per 100,000 per year vs. 2.8 per 100,000 for the entire state. The importance of an observation of elevated ALL in young females in Stearns County without a corresponding elevation in young males is hard to interpret. Nonetheless, from a public health surveillance perspective of identifying unusual occurrences of cancer through disease mapping, the Stearns County observation was an example of decreased sensitivity for the WSRT procedure. This was the only such example identified during the evaluation of the WSRT procedure.

## Discussion

There have been several methods developed to account for the loss of specificity due to multiple statistical comparisons [33]. Conventional approaches, such as the Bonferroni test of significance, are generally viewed as inappropriate in epidemiology as they diminish the ability to identify meaningful differences [26, 34, 35]. In the case of cancer mapping, there needs to be a middle ground due to the influence that it can have on public policy. Mapping of state cancer rates by county involves a large number of implied comparisons, and one of the goals of mapping these rates is the identification of unusual cancer occurrences that, when found, may have important public policy implications [15, 16, 36–38]. A recently established federal program, Environmental Public Health Tracking [39], is attempting to advance disease mapping and environmental correlations. A stated goal of this program is to recognize disease clusters in order to “understand the possible associations between the environment and the adverse health effects.”

This application of the WSRT procedure is another approach for assessing the significance of the difference between directly age-adjusted county cancer rates and the overall state age-adjusted rate. Traditional presentation of the pattern of county cancer rates occurs in several ways. Two of the most common are to portray the county rates as above or below the state average and to distribute the county rates by quartiles. These approaches have become a standard of practice [40]. The WSRT procedure does not replace these methods. The WSRT procedure is complementary to them, serving the objective of identifying meaningful significant differences between county and overall state rates without a major loss of sensitivity.

The large amount of random variation associated with the analyses of rates based on a small number of cancers raises concern over the precision of the estimates [41]. Several strategies are employed to address this problem. Indirect standardization as an age-adjustment method has been recommended for small counties where the age-specific rates are often quite variable and unreliable [42]. While this approach is useful in comparing a single county to the overall statewide rate, the results of indirect standardization may not be as useful when comparing multiple counties if there are large differences in the age structures of the populations. The indirectly standardized rate is weighted to the specific age distribution of the population of interest [43]. Since mapping county cancer rates results in comparisons among the counties, indirectly age-adjusting cancer rates are not recommended as a method for creating state cancer (and other rate) maps [44].

The most common strategy to address the small number problem is to suppress analyses that are based on a number of occurrences judged to be too low to yield valid results. This number is not consistently defined. The Kentucky Cancer Registry uses 15 [45] and the National Center for Health Statistics recommends 25 as the minimum [46]. The National Program of Cancer Registries does not provide rates if the cell size is smaller than 16 or the population smaller than 50,000 people [47]. Important results from identifying and studying populations with low cancer incidence are well documented [48, 49]. Suppressing analyses of low cancer rates solely on the basis that they represent numbers that are considered too low to publish may result in missing useful insights [31]. Mapping important differences between low county cancer rates compared to the overall state rate can be facilitated by the WSRT procedure due to its decreased sensitivity to low rate instability.

The evaluation of the performance of the WSRT procedure was empirical and based on cancer incidence data from Minnesota. Population-based cancer incidence data in the United States are collected under similar protocols [50], and it is likely that the WSRT procedure would perform similarly in other states. However, determination of whether the performance of the WSRT procedure found in this evaluation can be generalized for other applications and for use in all states requires further investigation.

The computational algorithm for the WSRT procedure used in this evaluation was coded in FORTRAN—a mathematically based programing language. The translation of this extension of the Wilcoxon’s signed rank test into an executable algorithm was straightforward and can be incorporated as a FORTRAN (available from the first author upon request) or C subroutine, or it can be created within a SAS program. The simplicity of coding required for calculating the WSRT significance is an appealing attribute of this procedure.

As the number of observations increase, the precision of the estimates increase, often resulting in small differences becoming statistically significant. The WSRT procedure was not as greatly impacted by the large number effect but it is not immune to it. Identification and interpretation of important differences in cancer rates will always require skill and judgment. The WSRT procedure is an ally in this process as it reduces the number of cancer rates that need to be evaluated.

As illustrated throughout the evaluation of the WSRT procedure, the level of significance for differences in directly age-standardized rates, such as 0.05 or 0.01 is theoretical; each application will likely yield a computed significance that is different than the desired theoretical level of significance. For the purposes of cancer mapping, it is not the level of significance that is important, but the fact that the significantly different rates represent truly unusual occurrences. John Tukey recommended that for disease mapping, significant differences be described as “unusual occurrences.” The WSRT procedure provides a useful method to portray the differences in directly age-adjusted cancer rates occurring at the county level compared to the overall state rate as “unusually low,” “not unusual,” or “unusually high.”

## Appendix 1

### Example calculations for the nonparametric procedure (WSRT)

Consider the data in Table 5 which contains age-standardized male cancer rates for all cancers combined for twenty counties and the entire state (per 100,000 per year). These data are from the MCSS for the period 1988–2007. The standard error for each of the counties (se_*i*_) and the state (se) is also provided in Table 5. The twenty counties were a sample (small, medium, and large) of all 87 Minnesota counties used to illustrate the WSRT procedure. The steps are identical to those described in the text.

**Table 5:** Age-standardized rates and their standard errors for males, all cancers combined

County/region	Standardized rate	Standard error
Entire State	555.62	1.18
Aitkin	546.56	16.10
Anoka	583.60	6.51
Becker	585.57	13.48
Beltrami	569.83	13.55
Big Stone	598.71	27.58
Blue Earth	528.55	11.07
Cass	566.25	13.16
Faribault	534.10	16.23
Fillmore	526.72	14.54
Goodhue	520.73	10.99
Kittson	466.45	25.96
Le Sueur	524.68	14.53
Lincoln	483.79	23.02
McLeod	546.21	13.11
Olmsted	628.29	8.34
Scott	564.36	11.58
St. Louis	554.88	5.21
Washington	541.64	7.27
Wright	553.24	9.72
Yellow medicine	516.53	19.23

MCSS 1988–2007

*Step 1* Create the 20, 95% standard normal confidence intervals (CIL_*i*_, CIU_*i*_) for the difference between the county rate and the state rate (diff_*i*_) from the formula: diff_*i*_ ± 1.96 $({\text{se}}_{i}^{2} + {\text{se}}^{2} )^{{1/2}}$. For example, Aitkin County: diff_1_ = 546.56 − 555.62 = −9.06; $({\text{se}}_{1}^{2} + {\text{se}}^{2})^{1/2} = (259.21 +1.3924)^{1/2} = 16.14.$

The (CIL_1_, CIU_1_) is −9.06 ± 31.64 = (−40.70, 22.58). The diff_*i*_ and their corresponding (CIL_*i*_, CIU_*i*_) for all twenty counties are given in Table 6. Note that there are 11 occurrences that fall outside their respective 95% standard normal confidence intervals, that is, the confidence interval for the difference does not contain zero.

**Table 6:** Differences between county and state rate (diff_*i*_), lower and upper 95% standard normal confidence limits (CL), and significance (Sig)

County	diff_*i*_	Lower CL	Upper CL	Sig
Aitkin	−9.06	−40.70	22.58	2
Anoka	27.98	15.01	40.95	3
Becker	29.95	3.43	56.47	3
Beltrami	14.21	−12.45	40.87	2
Big Stone	43.09	−11.02	97.20	2
Blue Earth	−27.07	−48.89	−5.25	1
Cass	10.63	−15.27	36.53	2
Faribault	−21.52	−53.41	10.37	2
Fillmore	−28.90	−57.49	−0.31	1
Goodhue	−34.89	−56.55	−13.23	1
Kittson	−89.17	−140.10	−38.24	1
Le Sueur	−30.94	−59.51	−2.37	1
Lincoln	−71.83	−117.01	−26.65	1
McLeod	−9.41	−35.21	16.39	2
Olmsted	72.67	56.16	89.18	3
Scott	−10.74	−21.21	−0.27	1
St. Louis	8.74	−14.07	31.55	2
Washington	−13.98	−28.42	0.46	2
Wright	−2.38	−21.57	16.81	2
Yellow medicine	−39.09	−76.85	−1.33	1

*1* low,* 2* not significant,* 3* high

*Step 2* For twenty counties, *m* = 20 and *M* = *m*(*m* + 1)/2 = 210. From the 210 Walsh averages for the upper and lower confidence limits. For example, for *i* = 1 and *j* = 2, $W_{L}^{k} = ({\text{CIL}}_{i} + {\text{CIL}}_{j} )/2 = ( - 40.70 + 15.01)/2 = - 12.84$ (rounded to two decimal places) and $W_{U}^{k} = ({\text{CIL}}_{i} + {\text{CIL}}_{j} )/2 = (22.58 + 40.95)/2 = 31.76$. These entries can be found in the partial tables of the Walsh lower limit averages (Table 7) and Walsh upper limit averages (Table 8).

**Table 7:** Walsh lower confidence limits averages (*M* = 210)

−40.70	−12.84	−18.64	−26.57	−25.86	−44.80	−27.98
−47.06	−49.10	−48.63	−90.40	−50.11	−78.85	−37.96
7.73	−27.39	−30.96	−34.56	−31.14	−58.78	15.01
9.22	1.28	2.00	−16.94	−0.13	−19.20	−21.24
−20.77	−62.55	−22.25	−51.00	−10.10	35.59	0.47
−3.10	−6.70	−3.28	−30.92	3.43	−4.51	−3.79

First 42 of the 210 Walsh lower limit averages

**Table 8:** Walsh upper confidence limits averages (*M* = 210)

22.58	31.76	39.53	31.72	59.89	8.67	29.55
16.48	11.14	4.68	−7.83	10.11	−2.04	19.49
55.88	27.07	11.16	11.52	19.70	10.63	40.95
48.71	40.91	69.07	17.85	38.74	25.66	20.32
13.86	1.36	19.29	7.15	28.67	65.06	36.25
20.34	20.70	28.88	19.81	56.47	48.67	76.83

First 42 of the 210 Walsh upper limit averages

*Step 3* Rank the lower and upper 210 Walsh averages in numeric order.

*Step 4* Calculate $C\alpha = \frac{m(m + 1)}{4} - Z_{(\alpha /2)} \left[{\frac{m(m + 1)(2m + 1)}{ {24} }}\right]^{1/2}$. For *m* = 20 and *Z*_(*α*/2)_ = 1.96, *Cα* = 52, rounded to the nearest integer and *M* + 1 − *Cα* = 159; *M* = 210.

*Step 5* The joint confidence interval is $\left( {W_{L}^{52},\quad W_{U}^{159} } \right)$. The 52nd ordered Walsh average is −55.45 and the 159th ordered Walsh average is 35.27. Thus, (−55.45, 35.27) is the joint confidence interval for the diff_*i*_ when zero lies outside the (CIL_*i*_, CIU_*i*_), *i* = 1, 20.

*Steps 6a and b* Compare each (CIL_*i*_, CIU_*i*_) in Table 6 to zero. If the (CIL_*i*_, CIU_*i*_) contains zero, the difference is considered nonsignificant. For Aitkin County, this interval contains zero. No further evaluation is required and comparison to the WSRT confidence interval, $\left( {W_{L}^{52} ,\quad W_{U}^{159} } \right)$, is not needed. The WSRT evaluation of the statistical significance of the difference is also taken as not unusual.

For Anoka County, the difference in age-standardized rates was 27.98. The corresponding (CIL_*i*_, CIU_*i*_) does not contain zero, and comparison of the Anoka County/State difference in rates to the WSRT confidence interval is appropriate. Since 27.98 is contained in the interval (−55.45, 35.27), the WSRT evaluation of significance is that the Anoka County rate was not unusual.

For Olmsted County (Rochester), diff_*i*_ = 72.67 and the 95% standard normal confidence interval, (56.16, 89.18), does not contain zero. This difference is also larger than the upper limit of (−55.45, 35.27) and the WSRT significance test indicates an elevated (unusually high) rate for Olmsted County.

Table 9 contains a summary of the 95% standard normal level of significance and the significance determined by the WSRT procedure. Eleven (8 low, 3 high) of the county rates were statistically different than the state rate as zero lies outside their respective 95% standard normal confidence intervals (Table 6). Only three (2 low, 1 high) were statistically unusual based on the WSRT procedure. This reduction in the number of significant differences is a salient feature of the WSRT procedure.

**Table 9:** 95% standard normal (SN) and WSRT determination of statistical significance for cancer rate data in Table 5

County	95% SN^a^	WSRT^b^
Aitkin	2	2
Anoka	3	2
Becker	3	2
Beltrami	2	2
Big Stone	2	2
Blue Earth	1	2
Cass	2	2
Faribault	2	2
Fillmore	1	2
Goodhue	1	2
Kittson	1	1
Le Sueur	1	2
Lincoln	1	1
McLeod	2	2
Olmsted	3	3
Scott	2	2
St. Louis	1	2
Washington	2	2
Wright	2	2
Yellow medicine	1	2

^a^ *1* Low,* 2* Not significant,* 3* High

^b^ *1*  Unusually low,* 2* Not unusual,* 3* Unusually high

## Appendix 2

See Table 10.

**Table 10:** List of 32 cancers used in the evaluation of the WSRT procedure

All types combined	Liver
Bone	Melanoma of the skin
Brain and nervous system	Mesothelioma (all sites)
Breast	Myeloma
Cervix	Non-Hodgkin lymphoma
Colon and rectum	Oral cavity and pharynx
Hodgkin lymphoma	Ovary
Kaposi sarcoma (all sites)	Pancreas
Kidney	Prostate
Larynx	Soft tissues (including heart)
Leukemia (all types)	Stomach
Acute lymphoid leukemia	Testis
Chronic lymphoid leukemia	Thyroid
Acute myeloid leukemia	Urinary bladder
Chronic myeloid leukemia	Uterus
